# Tissue microarrey: a potential cost-effective approach for mismatch repair testing in colorectal cancer

**DOI:** 10.1186/s12876-022-02573-7

**Published:** 2022-12-08

**Authors:** Shai Farkash, Naama Schwartz, Natalia Edison, Sophia Greenberg, Hila Belhanes Peled, Wail Sindiany, Judit Krausz

**Affiliations:** 1 Pathology Department, Emek Medical Center, Afula, Israel; 2 School of Public Health University of Haifa, Haifa, Israel

**Keywords:** Colorectal cancer, Mismatch repair, Tissue microarray, Digital image analysis

## Abstract

**Background:**

Deficiencies in Mismatch Repair (MMR) proteins are one of the major pathways in the development of colorectal cancer (CRC). MMR status evaluation is recommended in every new CRC patient. However, this is not fully implemented due to high costs. Tissue microarray (TMA) enables allocating tissue cores from few specimens to a single paraffin block. The primary objective of this study was to evaluate the accuracy of TMA MMR immunohistochemistry (IHC) compared to whole slide. The secondary objective was to evaluate and validate automatic digital image analysis software in differentiating pathological and normal TMA cores.

**Methods:**

Pathological cores were defined if at least one MMR protein was unstained. Tumoral and normal tissue of 11 CRC patients with known MMR status was used to obtain 623 TMA cores. The MMR staining of each core was evaluated by a pathologist and compared to the whole slide result. Digital analysis software by 3DHistech Ltd. was used to identify cell nucleus and quantify nuclear staining in 323 tissue cores. To identifying pathological tissue, cores the cohort was divided into a test (*N* = 146 cores) and validation sets (*N* = 177 cores). A staining intensity score (SIS) was developed, and its performance compared to the pathologist review of each core and to the whole slide result.

**Results:**

Compared to the whole slide, the pathologist’s assessment had 100% sensitivity (n/*N* = 112/112) and 100% specificity (n/*N* = 278/278) with 95% lower limit of 97 and 99% respectively. The area under the receiver operating characteristic (ROC) curve of SIS was 77%. A cutoff of 55 was obtained from the ROC curve. By implementing the cutoff in the validation dataset, the SIS had sensitivity and specificity of 98.2% [90.1–100%] and 58.5% [49.3–67.4%] respectively.

**Conclusions:**

The MMR status of CRC can be evaluated in TMA tissue cores thus potentially reducing MMR testing costs. The SIS can be used as triage indicator during pathologic review.

**Trial registration:**

Institutional ethical approval was granted for the performance of this study (Emek Medical Center Ethics ID: EMC-19-0179).

## Background

Colorectal cancer (CRC) is the third most frequently diagnosed cancer, with 694,000 deaths of 1.4 million prevalent cases each year worldwide according to GLOBOSCAN 2020. Unfortunately, the numbers of new cases seem to be rising and it is believed that by the year 2030 the global burden of colorectal cancer (CRC) is expected to increase by 60% to more than 2.2 million new cases and 1.1 million deaths [1, 2].

CRC can be either sporadic or hereditary. The majority of CRC cases are sporadic of which more than 80% arise from somatic mutations in the Adenomatous Polyposis Coli (APC) and additional 13% are attributed to deficiencies of the DNA Mismatch Repair (MMR) genes [3].

One of the most prevalent hereditary cancer prone syndromes is Lynch Syndrome (LS) also known as Hereditary Non-Polyposis Colon Cancer (HNPCC). The molecular basis of LS is deficiency in the MMR system [4].

The direct consequence of impaired MMR activity is microsatellite instability (MSI) – alteration in the length of tandem repeats within microsatellite regions [4]. Current laboratory assays for MSI and MMR include either a polymerase chain reaction (PCR) or an immunohistochemical (IHC) panel which demonstrates absence of 1 of 4 MMR enzymes MLH1, PMS2, MSH2,MSH6 [5].

It is estimated that of all new cases of colorectal cancer, 3% are attributable to LS [6].

The LS is characterized by predisposition to certain types of cancers, of which CRC and endometrial cancer (EC) are the most common. Among patients who have the LS, CRC tend to occur at younger age compared with patients with sporadic CRC (45 to 60 vs. 69 years of age) [7].

In the colon, LS associated cancers usually manifest as right sided tumors with a propensity for synchronous and metachronous CRC. Histologically, these tumors can present with poorly differentiated histologic features which may include mucinous features or a medullary growth pattern. These tumors tend to be infiltrated by lymphocytes which can be found between the cancerous glands [8–10].

Colorectal cancers, either sporadic or hereditary, which harbor a deficiency in DNA MMR mechanism, are diagnosed at early stages, have lower metastatic potential have better prognosis and higher disease free survival (DFS) when compared to proficient MMR tumors (pMMR) [11] Furthermore, the disease free survival of Stage III patients receiving the FOLFOX (fluorouracil, leucovorin, and oxaliplatin) therapy protocol was significantly longer in patients with deficient MMR (dMMR) CRC [12].

As mentioned above, MMR deficient CRC is characterized by increased density of tumor-infiltrating lymphocytes. This property makes these cancers possible candidates for immunotherapy with checkpoint inhibitors. Indeed, the anti–programmed death 1 (PD-1) antibodies pembrolizumab and nivolumab have been evaluated in patients with dMMR metastatic colorectal cancer in whom previous treatment with cytotoxic agents had failed. Pembrolizumab monotherapy and nivolumab monotherapy resulted in objective response rates that ranged from 31 to 52% (median follow-up time, 12 to 12.5 months) that were durable; similar responses were achieved in patients with LS associated CRC compared with non-LS CRC patients [13–15].

Therefore, identifying patients with deficient MMR CRC (either sporadic or hereditary) has a prognostic value and may be beneficial in tailoring a more suitable therapy for their disease.

Recent guidelines supported by multiple organizations including the Collage of American Pathologists (CAP) and the National Comprehensive Cancer Network (NCCN) have recommended the universal testing of all newly diagnosed CRC cases for deficient MMR or MSI in order to identify LS associated CRC cases [16, 17]. However, most CRC cases are not evaluated for MMR status because of the high costs associated with universal testing [18].

Tissue Micro Array (TMA) is an automatic system which enables allocating a few dozen tissue samples from their original standard paraffin blocks to a single TMA paraffin block, and subsequently to section and stain those samples simultaneously. In other words, as each standard paraffin block represent an individual patient, one TMA paraffin block represents many patients. Therefore, this method can be used with the potential benefit of significant reduction in cost and time [19].

Understanding the tumoral MMR status is of utmost importance not only for identifying patients with the LS or other MMR related familial CRC cases (and their family members), it is also important for accurate prognostication and for tailoring the best treatment to the patient. Indeed, the NCCN and the CAP recommend this test for every new case of CRC. With regard to testing MMR status in every new case of CRC, Israel is no different from other countries and the guidelines are not followed due to the high costs of the test.

The primary objective of this study is to demonstrate the accuracy of using TMA core evaluation compared to whole slide (i.e., ground truth). For this aim, a comparison between the core and the slide MMR status will be performed. As there are four proteins results per core, it was determined that the core had pathological finding if at least one of proteins was not stained. In case all of the proteins were stained, the core was classified as normal.

The secondary objective of this study is to evaluate and validate the automatic digital image analysis QuantCenter software by 3DHistech LTD. in differentiating pathological and normal cores. MMR proteins are stained by IHC and the software, which identify the nucleus, produces an automatic H-score for the nuclear staining of MMR protein. However, little is known about this score and it is very challenging to interpret its results. Also, the score is continuous, and no cutoff values were proposed. Thus, it is impossible to utilize this score in practice. Therefore, this study will propose a new staining score and examine both scores (H-score and the new staining score) for cutoff values to determine a pathological result as defined above.

## Methods

### Clinical specimen collection

All of the specimens (tumor and matched peritumoral tissue) were obtained from patients that were diagnosed with CRC at Emek Medical Center during 01.01.2012–30.12.2019. Only patients who had their tumoral tissue diagnosed for MMR status by immunohistochemistry (IHC) were included in this study. A cohort of 11 cases of colorectal cancer was collected from the department of diagnostic pathology patient data base of which 3 were dMMR. Tissue cores were obtained from the original tissue blocks and from tissue stocks kept our center. Because the study evaluates TMA as a potential technique for assessing MMR IHC staining, we used cores with normal colonic mucosa as well as cores with tumoral tissue. The tissue cores were stained for MMR by immunohistochemistry. Sufficient tissue was kept for possible future medical analyses.

### Tissue micro array construction

All tissue blocks were constructed by using a TMA grand master system (3DHistech Ltd., Budapest, Hungary). Each TMA block was constructed as follows: Each case underwent precise evaluation by a pathologist for the number of cores which could be extracted from it. Up to 60 2 mm cores were placed in each TMA block to form a total of 18 TMA blocks. The first section taken from each TMA block was used for H&E staining in order to evaluate each core for tumoral tissue and for quality assessment. Four additional sections were then cut and used for IHC staining for each one of the MMR proteins. Each TMA slide was than digitalized with Pannoramic MIDI Scanner (3DHistech Ltd., Budapest, Hungary) and the scanned virtual slides were evaluated by a pathologist.

### Immunohistochemical staining

For IHC staining, formalin-fixed 4-μm paraffin-embedded sections were mounted on Surgipath™ X-tra™ adhesive precleaned micro slides (Leica microsystems) and processed using an automated immunostainer (Benchmark-ultra, Ventana Medical System). Mismatch Repair (MMR) Protein Immunohistochemistry was performed using automated immunostainer (Ultra, Ventana Medical Systems) with the following antibodies: MSH2 (G219–1129), MLH1 (M1), MSH6 (44), PMS2 (EPR3947) by Ventana Medical Systems LTD, using standard protocols and procedures as indicated by the manufacturer. Visualization of the bound primary antibodies was performed using the opti-View DAB detection kit (Ventana Medical System). Sections were then counterstained with Gill’s hematoxylin, dehydrated and mounted for microscopic examination. All the slides were scanned by Pannoramic MIDI Scanner (3DHistech Ltd., Budapest, Hungary) and reviewed with Case Viewer software (3DHistech Ltd.).

Three hundred thirty-seven cores that were obtained from tissue stocks which were kept in formaldehyde showed lack of PMS2 nuclear staining due to over fixation. These cores were excluded from the study.

### Pathological assessment

The nuclear staining of MMR proteins was evaluated in both normal mucosa and CRC tumoral tissue. A cut section from a lymph node was used as positive control as well lymphoid follicles and stromal cells as internal controls. The pathologist who reviewed the immunostaining of the tissue samples was blinded to the MMR status of the patient from which the core was obtained. Stained slides and individual cores were scored as either stained (showing nuclear staining in at least some tumor cells) or unstained.

### Image color intensity categorization of MMR-stained TMA cores by a color indicator (including cutoff)

In order to assess the level of MMR proteins staining intensity 3DHISTECH Ltd. QuantCenter software was used. This sofware has a NuclearQuant feature that uses an algorithm which identifies the cell nucleus and calculates the intensity of the brown color which is considered as a positive staining by immunohistochemistry (IHC). The NuclearQuant module automaticaly calculates a histoscore entitled “H-score”. As mentioned earlier, little is known on the H-score formula. One study, by Orsolya Matolay et al. stated that the H-score is “using individual pixel intensity levels and the total area of pixels” [20]. Therefore, it was unclear how to analyze and interpret its results. Also, no cutoff values were proposed for practical use of this score. As a result, a new proposed staining intensity score was formulated. The new staining intensity score was composed of the automatic staining intensity components. The 3DHISTECH Ltd. QuantCenter software produces several average staining scores and for the new staining score the “Average positivity of Strong positive” was considered as the average staining score. As each core could have several focal areas of staining, the new score had to weigh its relative representation within the core. For example, if a core had a small area that had strong staining, but most of the core had no staining, the “Average positivity of Strong positive” would have misled the reviewer to think that the core was stained when in fact only a very small fraction of the core was stained. Thus, the new score included the staining evaluation in relation to the staining area in the core. The relative stained area of a core was determined with the relative amount of the stained pixels out of the entire core pixels:

$$Core\ section\ staining\ intensity\ score=B\ast W$$

$$W=\frac{\# stained\ pixels\ in\ a\ specific\ core\ section}{\# total\ pixels\ in\ a\ specific\ core\ section}$$

Where *B* is the core section’s specific “Average positivity of Strong positive” component and *W* represent the relative stained area in a core section of the specific MMR protein. Eventually, as the unstained enzyme represents a pathological result, and higher values of the new score represents higher staining intensity of the enzyme, the core section’s minimum staining intensity score was chosen as the representative staining intensity score of the specific core section.

### Statistical analysis

For each core, a pathological result was determined if at least one of the 4 MMR proteins was not stained according to the pathologist’s evaluation. The classification of each core was than compared to the whole slide pathologic assessment (ground truth). The per core sensitivity and specificity were presented with 95% confidence interval (95%CI).

The cohort of tissue cores was randomly divided into two separate databases. The 1st database was used to explore the staining intensity accuracy in classifying cores with and without staining according to the pathologist’s evaluation. For that aim, a receiver operator characteristic (ROC) curve was estimated with 95%CI. Based on the ROC curve, a cutoff value was selected for the best sensitivity and specificity in differentiating cores with or without staining. The same process was performed for the automatic H-score and the two ROC curves were compared [21].

The 2nd database was used to validate the performance of the staining intensity score and the H-score cutoff values, by presenting the sensitivity and specificity with 95%CI.

Finally, the staining intensity score and the H-score cutoff values were also compared to the whole slide ground truth result, by presenting the sensitivity and specificity with 95%CI.

## Results

**Fig. 1:**
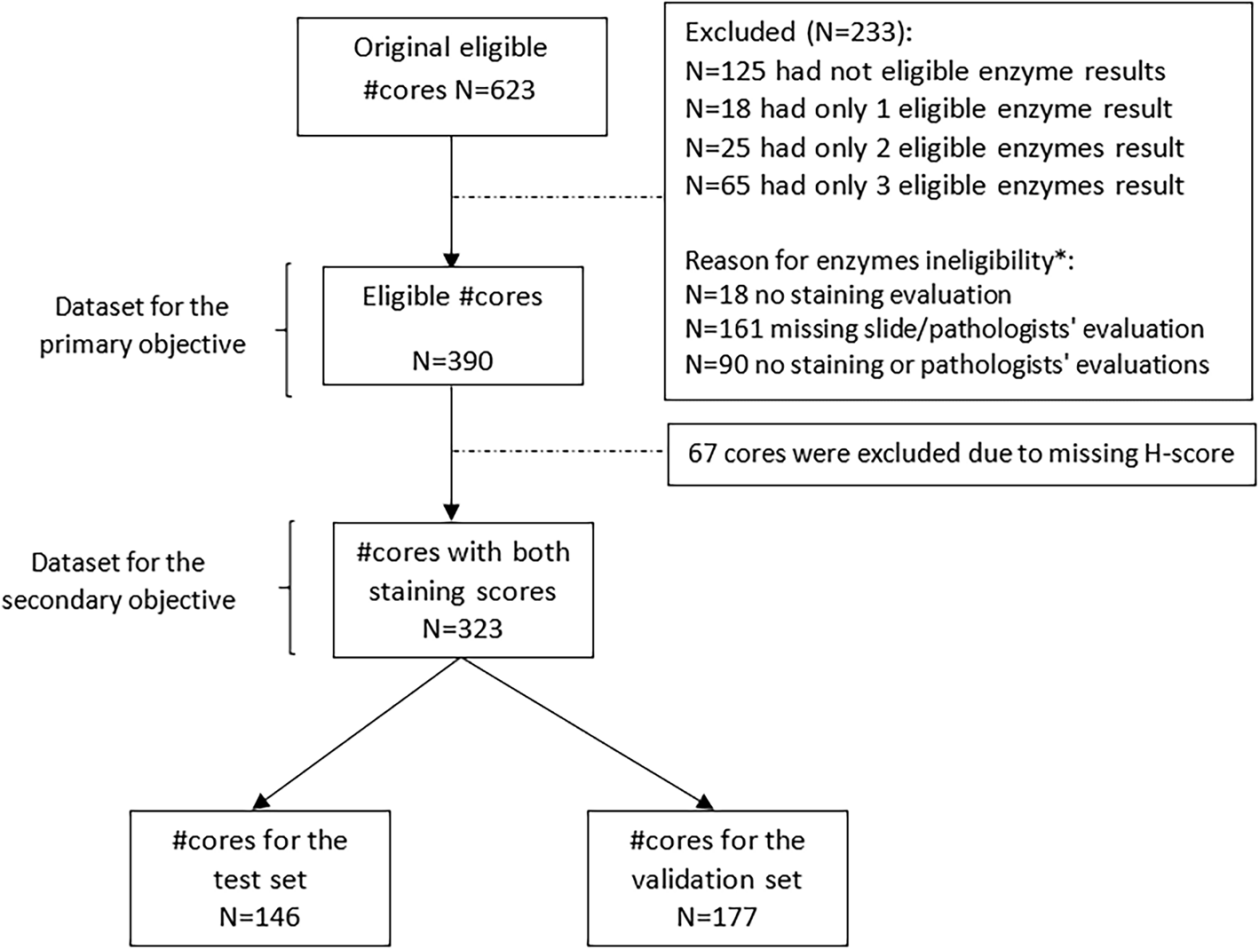
Study flow chart

Inorder to test the primary objective this study included 390 tissue cores and 323 to test the secondary objective (Fig. 1).

**Fig. 2:**
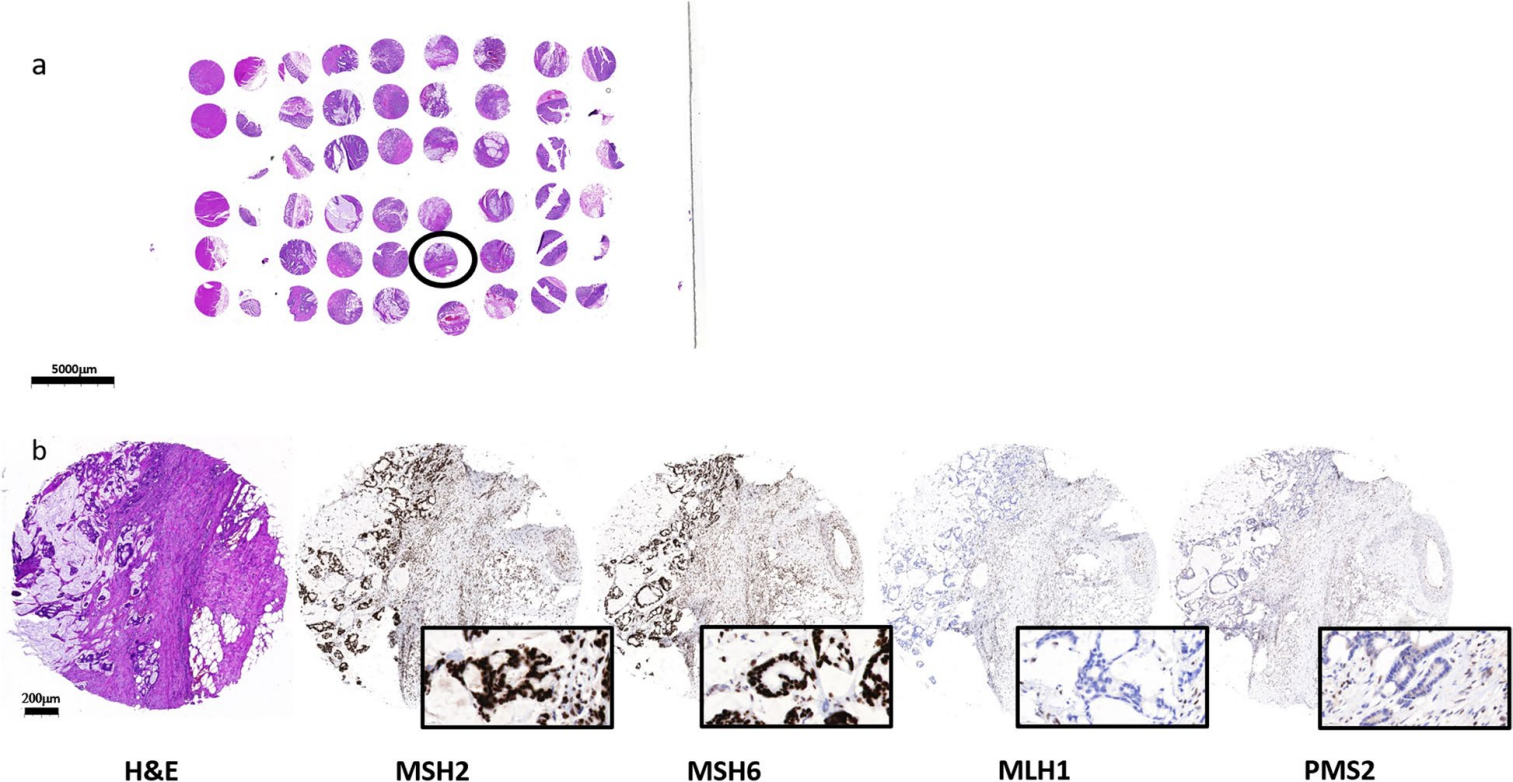
A representative TMA slide (**a**) and a core (encircled in 2a) showing colorectal cancer with its corresponding MMR stained (MSH2 and MSH6) and unstained sections (MLH1 and PMS2). The insets show the positive and negative nuclear staining (**b**). Magnification × 0.5 fold, scale bar = 5000 μm; × 4 fold, scale bar (black line) = 200 μm; X74.8 fold scale bar (black line) = 20 μm

A representative TMA slide and representative MMR stained and unstained cores are shown in Fig. 2.

### TMA accuracy measurements of IHC for MMR proteins

The ability to determine MMR status was not compromised when evaluated in TMA tissue cores compared to whole slide sections.

During a routine MMR status assessment, the patient’s tumor is stained for all four MMR proteins. If at least of these proteins does not show nuclear staining of tumor cells, the test is considered pathologic and the tumor potentially dMMR. Therefore, when evaluating TMA core sections to their expected whole slide results we defined a pathologic outcome if at least one of the MMR proteins had negative nuclear staining in each tissue core. For the purpose of this assessment only tissue cores with tissue sections of all four MMR proteins were included in the analysis.

Compared to the whole slide, the pathologist’s assessment had 100% sensitivity (n/*N* = 112/112) and 100% specificity (n/*N* = 278/278) with 95% lower limit of 97 and 99% respectively.

### Categorization of MMR-stained TMA cores by a color indicator

Digital image analysis was used to identify cores with at least one unstained MMR protein. For this purpose, the sensitivity was defined as cores sections which did not show nuclear staining and the specificity as cores section that showed nuclear staining. The staining intensity score was compared to the H-score which was calculated by 3DHistech Ltd. NuclearQuant software.

This analysis was performed by using two levels of comparison:

1. Core level comparison – staining intensity score and H score results of each core were compared to the staining result of each core.
2. Whole slide comparison – staining intensity score and H score results of each core section were compared to the whole slide ground truth staining result.

**Fig. 3:**
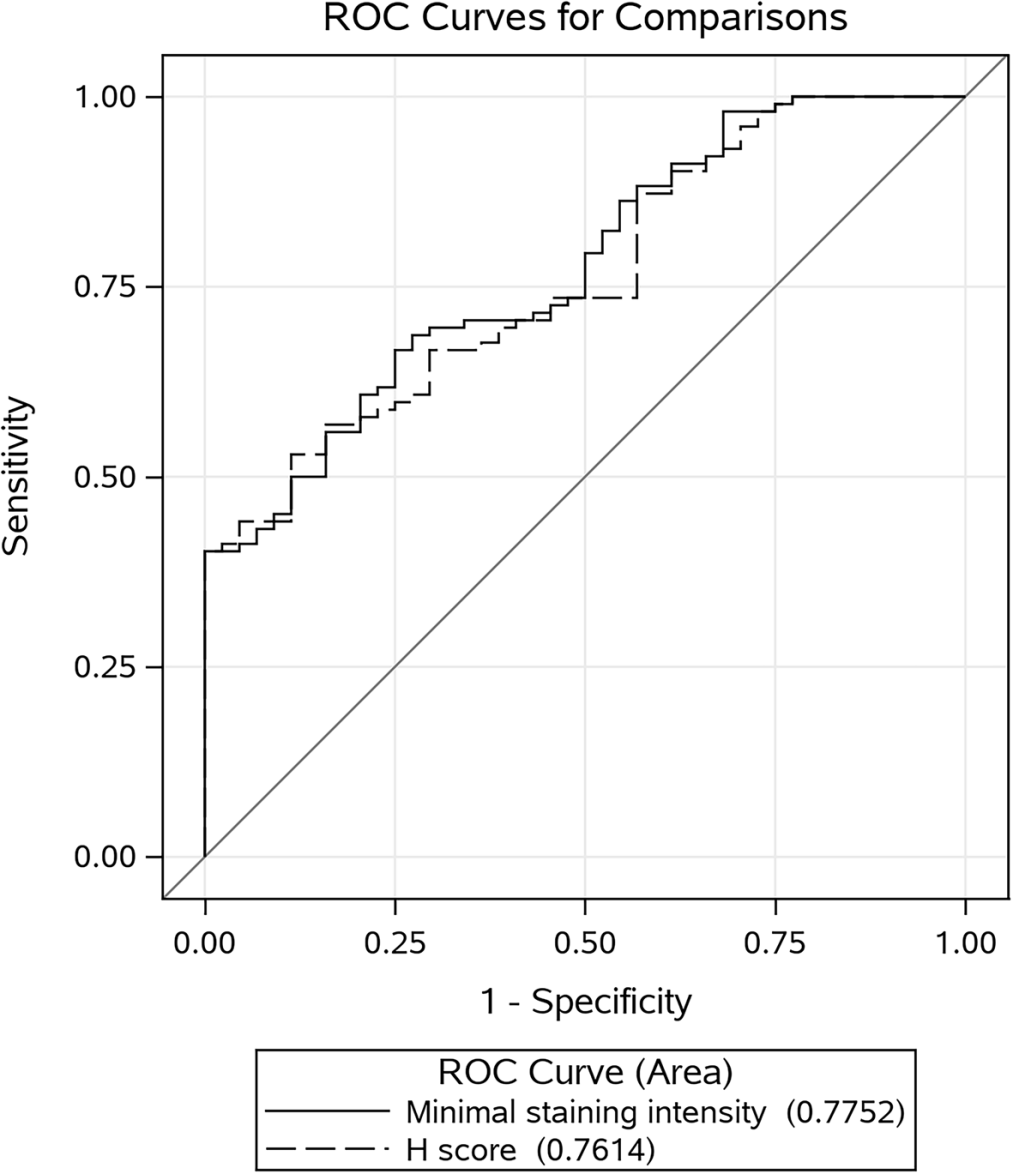
Receiver operator contributor (ROC) curves presenting the performance of the Staining intensity score and the H-score

Overall, the accuracy of the new proposed staining intensity score, as well as the H-score was identical and had fair accuracy - AUC for the staining intensity score and H-score was 77% (Fig. 3 ROC). Cutoff values based on the ROC curves were obtained and the accuracy measures for the test and validation datasets are presented in Table 1. The overall sensitivity of the per core staining classification according to the proposed cutoff values was good (validation set sensitivity lower 95%CI was more than 90%). Thus, these scores indication of pathological core may serve as a triage indicator for the pathologist’s review. When compared to the whole slide ground truth classification, the accuracy results were also good (Table 2). The specificity of the tool represents detection of non- pathological cores. Although the specificity is rather low the SIS or H-score are meant to be used as triage which will send pathological cores to the top of the list. Therefore, in this case, the specificity is not the main measure of interest and does not compromise the usefulness of the SIS. By estimating each MMR protein sensitivity independently, MSH6 and PSM2 had the largest contribution to the overall sensitivity.

**Table 1:** Sensitivity and specificity of the H-score and the staining intensity compare to the pathologist’s evaluation

	Cutoff	Test database (N = 146)		Validation database (*N* = 177)	
		n/N=Sensitivity [95%CI]	n/N=Specificity [95%CI]	n/N=Sensitivity [95%CI]	n/N=Specificity [95%CI]
H-score	168	37/44 = 84.1% [69.9–93.4%]	54/102 = 52.9% [42.8–62.9%]	53/54 = 98.2% [90.1–100%]	68/123 = 55.3% [46.1–64.3%]
Minimal staining intensity	55	37/44 = 84.1% [69.9–93.4%]	54/102 = 52.9% [42.8–62.9%]	53/54 = 98.2% [90.1–100%]	72/123 = 58.5% [49.3–67.4%]

*95%CI* 95%confidence intervals

**Table 2:** Sensitivity and specificity of the H-score and staining intensity score compared to whole slide classification

	Cutoff	Entire core sample (*N* = 323)	
		n/N=Sensitivity [95%CI]	n/N=Specificity [95%CI]
H-score	168	90/98 = 91.8% [84.6–96.4%]	122/225 = 54.2% [47.5–60.9%]
Minimal staining intensity	55	90/98 = 91.8% [84.6–96.4%]	126/225 = 56% [49.3–62.6%]

*95%CI* 95%confidence intervals

### Suggested workflow

**Fig. 4:**
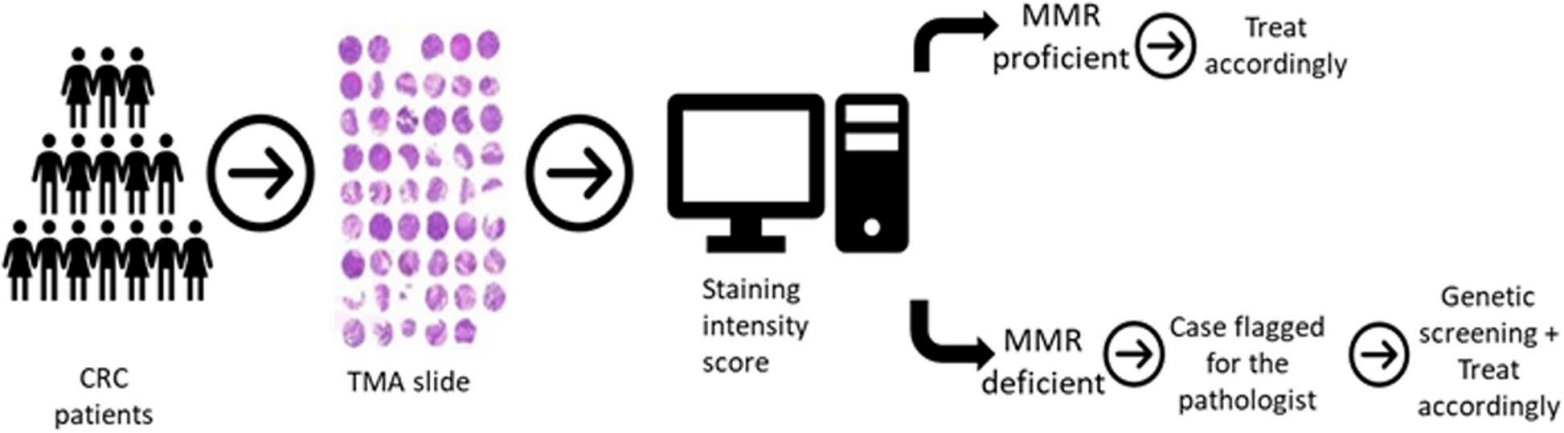
Suggested workflow

The work process suggested in this study is shown in Fig. 4: a TMA block with CRC tumor samples from few patients will be stained by IHC for MMR proteins. Each TMA core will be evaluated by a digital image analysis software using the staining intensity score. Every case will be automatically triaged based on the MMR staining status. Cores with at least one unstained MMR protein will be flagged for the pathologist to review. All the patients are eventually evaluated but reprioritization will be determined according to the MMR results.

## Discussion

### TMA accuracy measurements of IHC for MMR proteins

In this study it was established that TMA can be used for MMR evaluation with excellent accuracy. Hendricks et al. reported high concordence between whole slide sections and TMA in MMR staining of colorectal cancers although their concordance levels were 85, 95,88% for MLH1, MSH2 and MSH6 respectivly (PMS2 was not evaluated in this study) [22]. These results are slightly lower than what is reported in the current study. These differences can be attributed to the use of tissue blocks older than 10 years in the Hendricks et al. study. As stated above, in the current study the oldest tissue block dated 5 years back. Although it was not tested in the current study, it is possible that over the years the MMR immunostaining might be compromised even in paraffin embeded tissue. Furthermore, the conditions in which paraffin blocks are prepared and archived over the years may vary between different institutes and that may also contribute to the differences in MMR staining intessity.

A large scale study by A Nocito et al. compared between whole slide to TMA cores of 2317 bladder cancers. They tested whether TMA is a reliable tool in evaluating tumor grade and KI67 labaling index. Every individual association between grade or Ki67 and tumor stage or prognosis that was observed in large section analysis could be fully reproduced on all four replica TMAs [23].

A more recent study by Visser et al. tested the agreement for 15 biomarkers IHC stainings between TMA cores of differet sizes and whole slide sections of endometrial carcinoma. They evaluated specimans taken from 17 patients and tested an overall of 1020 core sections of 0.6 mm and 2 mm. This study showed that 2 mm tissue cores were more assessble than 0.6 mm cores. There was perfect agreement between MMR staining of 2 mm cores compared to whole slide sections. In the current study 2 mm cores were also used with excelent agreement compared to whole slide sections [24].

Geographic heterogeneity of MMR stainng within tumoral tissue is a well established phenomenon of this type of immunohistochemical staining and as reported by Greenberg et.al some of the MMR proteins such as MSH6 show higher levels of heterogeniety. The geographic heterogeniety of MMR staining can result in false positive results or even false negative results when evaluating whole slides [25]. Geographic heterogeniety of MMR staining poses an even greater challegne when using TMA because only a fraction of the tumor area is evaluated. Therefore, more than one tissue core should be sampled from each tumor. The optimal number of cores is under debate. Kyndi et al. reported that a single core seems to be sufficient, whereas Neves-Silva et al. reported that two cores per case is the optimal number with regard to tissue loss and agreement with whole slides. However, both groups did not evaluate MMR staining [26, 27]. The aim of this work was to evaluate the feasibility of TMA in MMR IHC staining. Although the geographic heterogeniety did not cause a significant decrease in TMA accuracy compared to whole slide sections, we believe that more than one tissue core should be used per patient in order to evaluate MMR status by the model suggested in this study.

At this point, it should be stressed that our analysis used a more practical approach by trying to detect a core for which at least one of the MMR proteins was unstained result. This approach in itself increased the level of accuracy but we used it as it corelates with the standard practice of MMR IHC analysis. We did not find previous reports which performed the comparison in this fashion.

### Computer based categorization of MMR-stained TMA cores by a color indicator (including cutoff)

The world of pathology undergoes tremendous changes of which the entry of digital pathology is one of the most significant. Digital pathology uses scaned slides for pathologic evaluation. This opens the door for automatic assesssment of slides by computer assisted diagnosis (CAD). Indeed, many studies examined the feasibility of incorporating CAD softwares into the routine pathologic examination [28–31]. Reports regarding CAD for MMR IHC analysis in TMA blocks are scarce. One large scale study by Echle et al. analyzed over 6000 CRC cases and used deep learning classifier to detect cases with which had dMMR or MSI. The classifier detected these cases using hematoxylin and eosin slides and was based on previous data regarding MMR status of CRC tumors in the training data set. The classifier had a mean area under the ROC curve of 92% [18]. Other studies used automated digital image analysis of KI67 levels and other proteins in TMA breast cancer tissue cores [31–33]**,** these studies show the advantages of TMA as a cost effective and as a standalone technique and further emphasize the advantages in combining it with automated digital image analysis software. Another study by Guy Nir et al. used automated digital image analysis to determine the grade of prostate cancer in 333 TMA tissue cores. Their classifier was shown to be within the interobserver variability between pathologists [34].

This study used NuclearQuant software (3DHistech Ltd. Budapest, Hungary), which can identfy cell nucleaus, quntify brown color intensity and amount of pixcels (brown and overall) in each core section. The readings of staining intensity were used to formulate the staining intensity score which takes into account the area of the core which is stained brown in addtion to color intensity.

The same practical approach of day to day routine MMR IHC testing was also applied for the staining intensity score and H-score analyses.

Therefore, as negative staining is sought for, in each core, the cutoff was applied on the section with the lowest score value (either staining intesity score or H-score) and if the score was lower than the cutoff, the MMR protein represented by this section was considered as negative and the core flagged as pathologic. By using this approach, both scores showed a fair level of performance with similar good sensitivity and rather low specificity. The high sinsitivity represents the ability of the scores to detect pathological cores, seperating them from the rest of the cores and sending them to the top of the line. The low specificity only means that some non- pathological, MMR profocient samples, will also be pushed to the top of the line. However, this is of minor importance because almost all MMR deficient cores will still be identified. In addtion, all the cores will be evaluated by a pathologist eventually.

It was Surprising that the cutoff values for each score were very different: 55 for the staining intensity score and 168 for the H-score. This implies that although both scores show identical performance, the scores are calculated differently. The 3DHISTECH H-score feature was also used by Orsolya Matolay et al. to quantify stainng intensity of carbonic anhydrase IX (CAIX) and CD30 in digitaly analysed Hodgkins lymphoma tissue sections. The H-Score proved to be valid compared to light microscopy “ground truth”. The group suggested explanation as to how the H-scores works resembles our appraoch for the staining intensity score [20]. However, the differences show that there are other aspects included in the H-Score calculation. Others, like Marcin Brown et al. showed high level of agreement between pathologic evaluation and the 3DHistech H-Score in identifying fibroblast growth factor receptor-2 (FGFR2) in breast cancer samples [28]. These studies are in agreement with the results of this study regarding H-Score performance.

This study demonstrated that by combining either score into an automated digital image analysis software it will be possible to differentiate tissue cores with MMR nuclear staining from unstained tissue cores.

The limitations of this study are the small number of patients (*n* = 11). Future studies should evaluate MMR IHC by TMA by using a per patient analysis. Another limitation of this study is the use of healthy colonic mucosa (in addiotn to tumoral colonic tissue). However, since the aim of this work was to evaluate TMA technique in the use of MMR protein IHC staining, we think that this limitation is of minior importance. Furthermore, because this study evaluated the use of TMA technique for MMR IHC, sections from healthy tissue were included as an internal quality control samples for TMA MMR staining.

As mentioned in Methods section, 337 cores were excluded from the study because of complete loss of PMS2 nuclear staining and decrease in nuclear staining intensity of MLH1, MSH2 and MSH6. All of these cores were obtained from tissue stocks which were kept in formaldehyde for long periods of time (at least 3 weeks). In contrast, tissue samples taken from the same patients at shorter periods of fixation (1–3 days) showed intact, strong nuclear staining of PMS2 and the rest of the MMR proteins. We attribute this observation to the duration of formalin fixation. Indeed, it is well established that many factors influence the results of MMR IHC staining such as fixation and oxidative stress. In fact, the variability in fixation of tumor tissue was referred to by Susan Richman [35], as “the single biggest problem in the assessment of MMR IHC“. Another study by Fadhil et al. showed that tissue fixation affects the PMS2 staining of CRC [35-37]. Therefore, the issue of tissue over fixation and its effect on MMR IHC should be addressed in future studies.

## Conclusions

Our results show that MMR IHC can be performed on TMA tissue blocks thus potentilay lowering the costs of the test per case as a number of cases can be evaluated on the same slide.

In a hospital settings, the pathologist has to review many cases with suspected colorectal cancer. Our suggested workflow can be used to prioritize those cases in which there is a potential abberant MMR stainng pattern (i.e a tissue core with one of the the MMR proteins showing negative nuclear staining) before evaluating the rest of the cases.

According to recent guidelines, every new case of CRC should be evaluated for MMR as part of LS screening. In reality only a minority of CRC cases is evaluated for CRC. In a recent study evaluating the benefits of reflex MMR testing in Ontarioi, Vanessa N. Palter et al. describes a framework for a LS reflex testing program according to which one of the main issues is centralization of testing which ensures consistency and quality assurance of testing [38]. For this matter, TMA could be an excellent platform for centralization of MMR testing as it lowers the costs and allows good quality assurance as many cases are tested simultaneously on the same slide.

To our knowledge this is the first time a workflow for routine MMR automated analysis is presented in TMA tissue cores. Further larger scale studies are needed to evaluate TMA based assessment of MMR IHC in CRC pateints and to evaluate the financial merit of this method. Other cancers should be considered as well in future studuies.

## Data Availability

The datasets generated during and/or analyzed during the current study are available from the corresponding author on reasonable request.

## Abbreviations

MMR: Mismatch repair
CRC: Colorectal cancer
SIS: Staining intensity score
TMA: Tissue microarray
IHC: Immunohistochemistry
ROC: Receiver operating characteristic
APC: Adenomatous Polyposis Coli
LS: Lynch Syndrome
HNPCC: Hereditary Non-Polyposis Colon Cancer
MSI: Microsatellite instability
PCR: Polymerase chain reaction
EC: Endometrial cancer
DFS: Disease free survival
pMMR: Proficient MMR
dMMR: Deficient MMR
CAD: Computer assisted diagnosis
CAIX: Carbonic anhydrase IX
FGFR2: Fibroblast growth factor receptor-2
